# Conditioned flight response in female rats to naturalistic threat is estrous-cycle dependent

**DOI:** 10.1038/s41598-023-47591-x

**Published:** 2023-11-28

**Authors:** Gyeong Hee Pyeon, Jaeyong Lee, Yong Sang Jo, June-Seek Choi

**Affiliations:** School of Psychology, Korea University, Seoul, Republic of Korea

**Keywords:** Psychology, Emotion

## Abstract

Despite the prevalent expression of freezing behavior following Pavlovian fear conditioning, a growing body of literature suggests potential sex differences in defensive responses. Our study investigated how female defensive behaviors are expressed in different threat situations and modulated by the estrous cycle. We aimed to compare freezing and flight-like responses during the acquisition and retrieval of fear conditioning using two distinct unconditioned stimuli (US) in two different spatial configurations: (1) electrical footshock (FUS) in a small, conventional enclosure with a grid floor, and (2) a predator-like robot (PUS) in a spacious, open arena. Fear conditioning with FUS showed no substantial differences between male and female rats of two different estrous cycles (proestrus and diestrus) in the levels of freezing and flight. However, when PUS was employed, proestrus female rats showed significantly more flight responses to the CS during both acquisition and the retrieval compared to the male and diestrus female rats. Taken together, our findings suggest that hormonal influences on the choice of defensive strategies in threat situations are significantly modulated by both the type of US and the spatial configuration of the environment.

## Introduction

Anxiety and fear-related disorders are more common in women than in men, with a lifetime prevalence nearly twice as high^1,2^. Therefore, it is crucial to understand the fundamental disparities underlying fear and anxiety to develop more comprehensive and effective treatments for both sexes^3,4^. However, a substantial gap in our understanding stems from the fact that the majority of basic research in this field has predominantly involved male animals, resulting in a scarce data from female rats^5^. This is further complicated by mixed results from studies involving female rodents. Some studies suggest that females exhibit more active defensive behaviors such as flight responses^6,7^, while other studies have observed the opposite, where females demonstrate more passive defensive behaviors than males^8^. Adding to this complexity, a number of studies have reported no sex differences in the levels of either freezing or flight responses^9,10^. Therefore, despite the growing inclusion of female rats in recent research, there is a clear need for more systematic analyses that specifically investigate sex differences in animal research^11,12^.

Pavlovian fear conditioning serves as a standardized laboratory procedure for investigating threat processing^13–15^. In this paradigm, animals are repeatedly presented with a neutral conditioned stimulus (CS)—typically a tone—and an aversive unconditioned stimulus (US), such as an electrical footshock. The footshock immediately produces reflexive jumping followed by freezing, a common defensive response characterized by a robust state of immobility^16–18^. Interestingly, the choice of defensive strategies, including freezing, depends on contextual factors, one of which is perceived distance from the threat^19^. Although the footshock, delivered through a metal grid, simulates a painful encounter with a predator^20^, it remains invisible to the animals, making the distance to the threat indeterminable. As animals cannot predict the distance from this invisible threat, freezing becomes the most probable defensive behavior in the fear conditioning paradigm. However, survival in natural environments requires more flexible defensive strategies in the face of dynamic threat situations. Given this, recent studies have incorporated ecological threat paradigms, including real predators^21,22^ or predator-like robots^23–25^, to explore defensive behaviors beyond freezing. Thus, these ecological paradigms could potentially resolve the ongoing controversy surrounding the existence of sex differences in defensive behavior.

To address this issue, this study aimed to probe the potential influence of sex on defensive behaviors, with an emphasis on how the type of US and the extensive environment of a naturalistic setting shape these variations. For this purpose, we initially employed conventional fear conditioning using an electrical footshock as US (FUS). We then employed a fast-approaching predator-like robot as a new type of US (PUS), allowing animals to visually and audibly detect the impending threat, and observed any changes in defensive behaviors. In addition, to investigate the role of hormonal influences, we monitored the naturally cycling estrous stages in females. With an aim to clarify the discrepancies in existing findings about female defensive behavior, our experiments suggest that there are sex differences in defensive behavior in response to CS and the choice of defensive behaviors is influenced by the type of US and the spatial configuration of the environment where the US is presented. These findings highlight the necessity for careful consideration of these factors when studying sex differences in defensive behaviors.

## Results

### Cycle categorization based on nucleated epithelial cells and leukocytes

**Figure 1:**
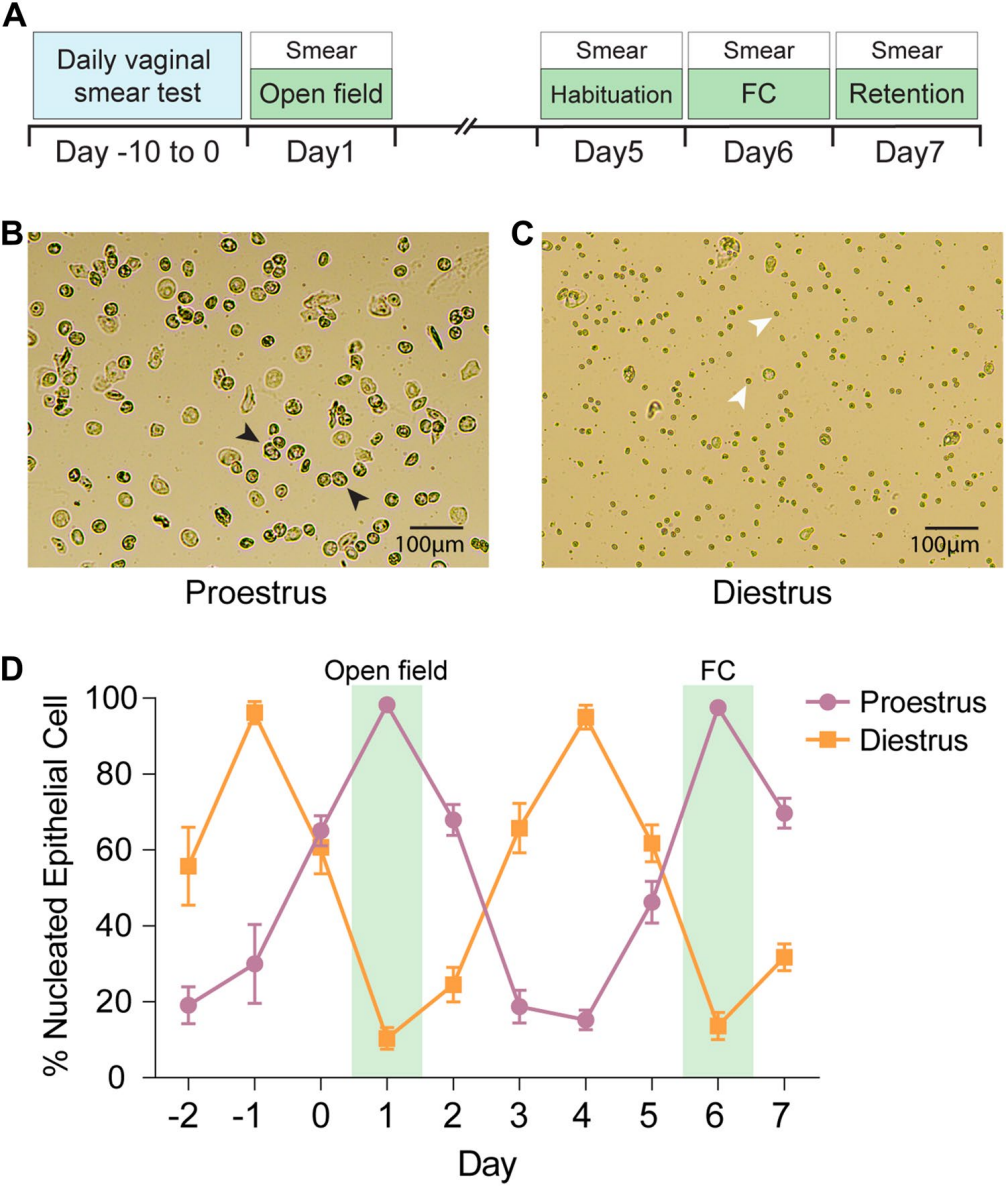
Experimental schedule and estrous cycle determination. (**A**) Procedures for fear conditioning (FC) experiments. (**B**,**C**) Photomicrographs of vaginal smears from rats showing two phases of the estrous cycle. (**B**) The proestrus phase was characterized by an abundance of nucleated epithelial cells (black arrows). (**C**) During the diestrus period, leukocytes (white arrows) were densely observed. (**D**) Representative day-to-day estrous cycles for proestrus (n = 9) and diestrus groups (n = 9) charted over 10 days based on the number of nucleated epithelial cells. The shaded green area represents the day when open field test and fear conditioning was conducted.

Instead of examining behaviors across all four stages of estrous cycle in female rats, we focused on the proestrus and diestrus because those two stages are the most dynamic periods of hormonal differences. Levels of ovarian hormones (estrogen and progesterone) peak during proestrus, while these hormones reach the lowest point during diestrus^26^. Proestrus was characterized by a predominance of nucleated epithelial cells, which are round or oval-shaped with centrally located nuclei within the cytoplasm (Fig. 1B; black arrows). Conversely, diestrus was marked by presence of leukocytes, which are small, round-shaped cells that lack visible nuclei when viewed at low magnifications (Fig. 1C; white arrows).

For accurate classification, rats were assigned to either proestrus or diestrus stages when at least 80% of cells in the smear tests corresponded to the specific cell type. To confirm at least two complete regular estrous cycles, considering the typical rat estrous cycle length of 4–5 days^27^, we monitored the ratio of epithelial cells and leukocytes for a minimum of 10 days. Figure 1D illustrates the resulting daily fluctuations in the cycle. In addition, in cases where the estrous cycle was unstable, we delayed behavioral tests until the peak occurrence of either the proestrus or diestrus stage was identified. To recap, once we observed the peak occurrence of either type of cells, we grouped the female rats into either proestrus or diestrus. Then, 1 h later, both these female groups, along with male rats, underwent behavioral testing.

### Heightened locomotor activity in proestrus females

**Figure 2:**
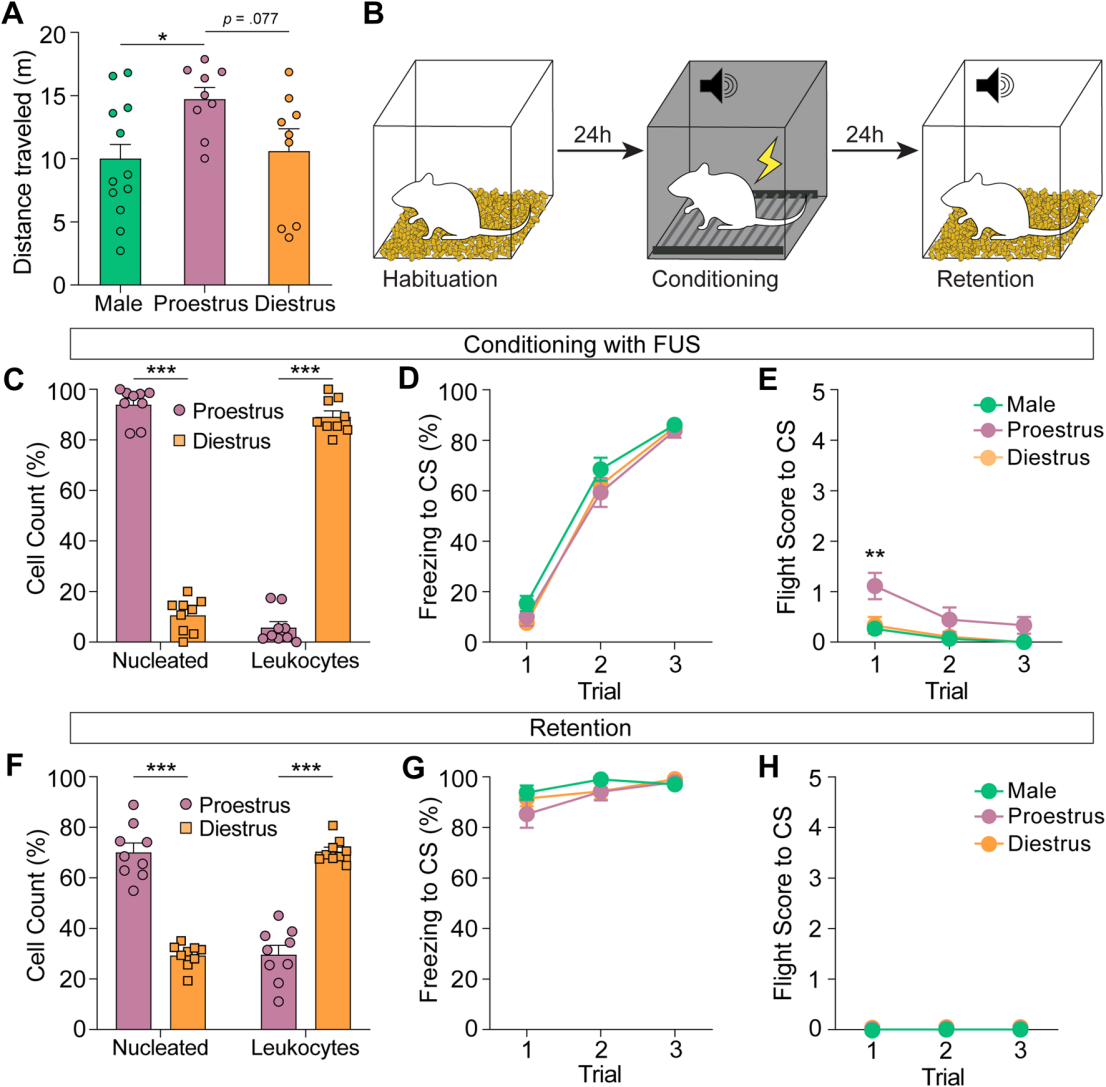
Fear conditioning with footshock US (FUS) induces predominant freezing response in rats. (**A**) Distance traveled in the open-field arena over 10 min. Proestrus female rats traveled more compared to both male and diestrus female rats. (**B**) A schematic diagram of fear conditioning protocol with FUS. (**C**) Categorization of the estrous cycle stages. Proestrus female rats showed a higher proportion of epithelial cells than diestrus females (*t*(16) = 26.40, *p* < 0.001). (**D**) Freezing in repones to the tone CS during conditioning. All three groups showed an equivalent progressive increase in freezing. (**E**) Flight scores to the CS during conditioning. Note that only the proestrus female rats displayed flight responses on the first trial. (**F**) Proportion of nucleated epithelial cells and leukocytes on the retention day. Proestrus female rats showed a higher proportion of epithelial cells than diestrus females (*t*(16) = 10.54, *p* < 0.001). (**G**) Freezing during the retention. All three groups showed an equivalent level of freezing. (**H**) Flight scores during the retention. None of the animals displayed flight responses. *p < 0.05, ***p < 0.001.

We allowed the male, proestrus and diestrus groups to freely explore the open arena for 10 min and assessed their general activity levels by measuring the distance each group traveled during this period. Notably, proestrus female rats moved a greater distance compared to other groups, resulting in a significant group difference (*F*(2,27) = 3.83, *p* < 0.05). Bonferroni pairwise comparisons revealed that proestrus female rats (n = 9) traveled significantly greater distance compared to male (n = 12; *p* < 0.05). Although the distance traveled by proestrus females was greater than that of diestrus females (n = 9), this difference was marginal (p = 0.077; Fig. 2A). This reveals the modulatory role of the female sex hormones in general activity.

### Fear conditioning with footshock as US (FUS) promotes freezing responses

To examine the potential modulation of defensive behavior by the estrous cycle, we conducted fear conditioning experiments with electrical footshock as US (FUS), and the protocol is illustrated in Figs. 1A, 2B. We ensured that female rats of two different estrous cycles reached peak levels of the corresponding cellular markers on the day of acquisition (Fig. 2C). Animals from all three groups received three pairings of CS (2 kHz, 80 dB, 10 s) and co-terminating FUS (0.6 mA, 1 s). There was a significant effect of trial (*F*(2,81) = 296.37, *p* < 0.001), indicating that all three groups showed progressive increase in freezing during the acquisition of the CS–FUS association (Fig. 2D). In addition, all three groups displayed a comparable level of freezing, as confirmed by no significant effect of group (*F*(2,81) = 2.29, *p* = 0.11), and there was no significant interaction between these factors (*F*(4,81) = 0.39, *p* = 0.82). We then analyzed the flight score, which encompasses rapid forward movements akin to running and vertical movements such as jumping (Fig. 2E), and separate measures quantifying each of these behaviors—fleeing and jumping—independently (Supplementary Fig. S1A and S1B). A two-way ANOVA showed significant effects of both group (*F*(2,81) = 12.65, *p* < 0.001) and trial (*F*(2,81) = 9.21, *p* < 0.001) on the levels of flight score, but no interaction between these factors was observed (*F*(4,81) = 1.25; *p* = 0.30). Planned comparison demonstrated significant differences between proestrus female rats and both male (*p* < 0.001) and diestrus female (*p* < 0.001) rats, but these differences were observed only on the first trial (Fig. 2E). Due to the absence of jumping behavior, significant values for fleeing behavior were identical to the flight score. Given that the responses on the first trial of conditioning occurred before the animals experience the shock, it is likely that the observed flight responses in proestrus female rats were due to the elevated baseline locomotor activity, as previously shown in the open field test (Fig. 2A).

Fear memory was tested 24 h after the conditioning by presenting three CSs. Recognizing the potential shift in the estrous cycle within a 24-h period, we collected and assessed vaginal samples 1 h before the retention test. Although there was a decrease in the specific cellular markers compared to the conditioning day, approximately 70% of these markers on the retention day aligned with their respective group (Fig. 2F). During the retention period, all animals dominantly displayed freezing responses across trials, with no significant differences in average freezing levels detected among the groups (*F*(2, 81) = 0.92, *p* = 0.40; Fig. 2G). As the animals primarily exhibited freezing behavior during the presentation of the CSs, no flight responses were detected in any of the rats (Fig. 2H). Moreover, the absence of initial flight responses in proestrus female rats during the retention further reinforces the notion that these flight responses in this experiment were not conditioned responses. Together, fear conditioning with footshock appears to primarily elicit freezing behavior in animals, regardless of sex and estrous cycle stages.

### Naturalistic threat promotes active defensive behavior in proestrus female rats

**Figure 3:**
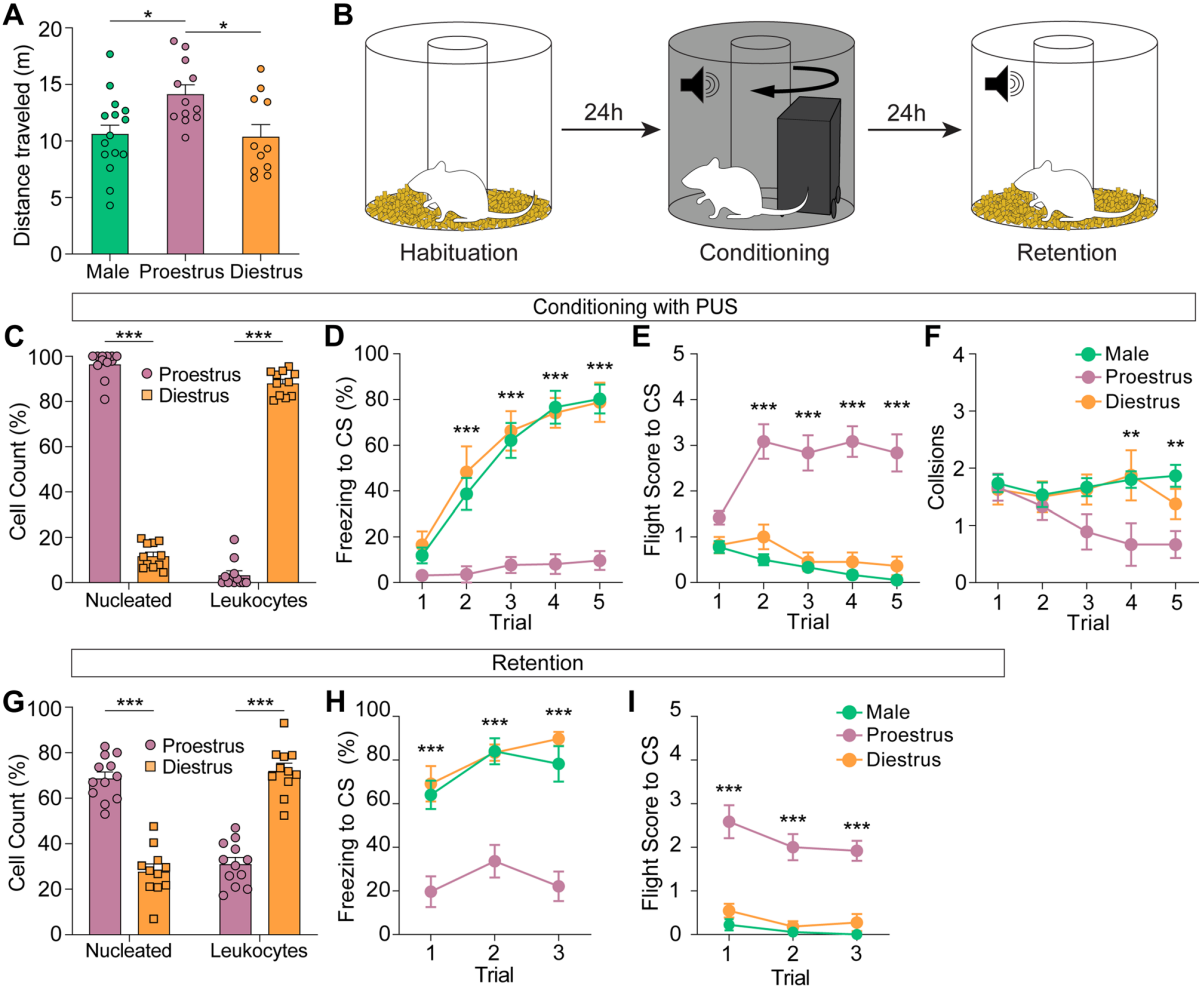
Fear conditioning with predator-like robot US (PUS) promotes conditioned flight responses in proestrus female rats. (**A**) Distance traveled in the open-field test. Consistent with the previous results from Exp. 1, proestrus female rats moved a greater distance compared to the other two groups. (**B**) Schematic of the fear conditioning protocol using PUS. (**C**) Cytological examination to determine the stage of the estrous cycle. Proestrus female rats showed a higher number of epithelial cells compared to diestrus female rats (*t*(21) = 34.90, *p* < 0.001). (**D**) Freezing to the CS during fear conditioning. Progressive increase in freezing was observed in both male and diestrus female rats, but not in proestrus female rats. (**E**) Flight scores to the CS. Proestrus female rats exhibited significantly higher number of flight responses compared to both male and diestrus female rats. (**F**) Number of collisions with the chasing robot. No group differences were found until trial 4. During trial 4 and 5, proestrus female rats collided less frequently compared to both male and diestrus female rats. (**G**) Proportion of nucleated epithelial cells and leukocytes on the retention day. Proestrus female rats showed a higher proportion of epithelial cells than diestrus females (*t*(21) = 9.73, *p* < 0.001). (**H**) Freezing during retention. Both male and diestrus females exhibited higher levels of freezing compared to proestrus females. (**I**) Flight scores during retention. Proestrus females exhibited a greater number of flight responses than the other two groups. *p < 0.05, **p < 0.01, ***p < 0.001.

To further investigate the influence of the estrous cycle on defensive behavior in more ecologically relevant fear paradigm, we employed a predator-like chasing robot US (PUS) and expanded the testing environment to allow more room for escape (Fig. 3B). The same conditioning schedule (Fig. 1A) was applied. Prior to the fear conditioning experiment, we classified a new set of female rats into either proestrus or diestrus, as previously described in Fig. 2C, and these animals were subjected to the open field test. Consistent with our previous results (Fig. 2A), we observed a group difference in distance traveled (*F*(2,35) = 5.56, *p* < 0.01), with proestrus female rats moving a greater distance compared to both male (*p* < 0.05) and diestrus female (*p* < 0.05) rats (Fig. 3A).

Subsequently, when the female rats reached their next cytological peak (Fig. 3C), male (n = 15), proestrus female (n = 12), and diestrus female (n = 11) rats underwent fear conditioning. During the acquisition, all three groups received five parings of CS and PUS (Fig. 3B). The predator-like robot, designed to simulate a predatory threat by chasing the animals at a speed of 0.7 m/s in an inescapable maze, was modeled after the design from a previous study^25^. There were significant main effects in both group (*F*(2,175) = 85.02, *p* < 0.001) and trial (*F*(4,175) = 23.58, *p* < 0.001) in freezing, as well as a significant interaction between these variables (*F*(8,175) = 4.50, *p* < 0.001; Supplementary Video 1 and 3). Post hoc analyses with Bonferroni adjustments indicated significantly lower levels of freezing in proestrus female rats compared to other groups on trial 2–5 (*p* < 0.001; Fig. 3D). These results might suggest an impairment in associative fear learning in proestrus female rats. However, we observed a substantial number of active defensive responses in proestrus female rats (Supplementary Video 2). To validate this, we quantified and analyzed the flight score across all three groups. A two-way ANOVA identified significant effects of both group (*F*(2,175) = 161.50, *p* < 0.001) and trial (*F*(4,175) = 2.46, *p* < 0.05), with a significant interaction between them (*F*(8,175) = 5.46, *p* < 0.001) on flight scores. Subsequent post hoc comparison revealed that flight scores from proestrus female rats were significantly higher compared to both male and diestrus female rats on trial 2–5 (*p* < 0.001; Fig. 3E). When examining flight behaviors separately, there was a significant effect of group (*F*(2, 81) = 9.21, *p* < 0.001), a marginal effect of trial (*F*(2, 175) = 2.27, *p* = 0.06), and a significant interaction between them (*F*(8, 175) = 4.35, *p* < 0.001). During trials 2–5, proestrus females exhibited a higher frequency of fleeing compared to other groups (*p* < 0.001; Supplementary Fig. S2A). For jumping, there were significant group differences (*F*(2, 175) = 51.93, *p* < 0.001), a marginal trial effect (*F*(2, 175) = 2.25, *p* = 0.07) and a significant interaction effect (*F*(8, 175) = 2.19, *p* < 0.05). Notably, jumping behavior was exclusive to proestrus females, demonstrating a higher frequency during trials 2–5 compared to other groups (*p* < 0.01; Supplementary Fig. S2B). In addition, after observing animals collide with the chasing robot, we analyzed the number of collisions during conditioning to examine group differences across trials. A two-way ANOVA revealed a significant group difference (*F*(2, 175) = 8.46,* p* < 0.001). However, there was no significant effect observed for trial (*F*(4, 175) = 1.10,* p* = 0.36), nor was there an interaction between group and trial (*F*(8, 175) = 1.69,* p* = 0.103). Planned comparison showed that while no group differences emerged until trial 3, the proestrus group had significantly fewer collisions on trials 4 and 5 (*p* < 0.01) compared to both male and diestrus female rats, which showed no difference between them (Fig. 3F). To determine whether collisions influenced the formation of defensive behaviors, we analyzed the correlation between the number of collisions and freezing (male: r = − 0.18, *p* = 0.53; proestrus: r = 0.44, *p* = 0.23; diestrus: r = − 0.08, *p* = 0.86) or flight (male: r = − 0.08, *p* = 0.78; proestrus: r = 0.01, *p* = 0.97; diestrus: r = 0.35, *p* = 0.40) in each group. The results suggest that collision had little contribution to shaping the defensive behavior. Therefore, the different number of collisions in proestrus rats might indicate that they preferred flight response in the same threat situation, reflecting the difference in motivation-to-action mapping due to the cycle difference.

On the retention day, a vaginal smear test was conducted on the female rats to verify that their estrous cycles remained consistent with their initial group assignments (Fig. 3G). All three groups exhibited persistent defensive behaviors consistent with those observed during the acquisition phase. A two-way ANOVA revealed significant effects of both group (*F*(2,105) = 53.95, *p* < 0.001) and trial (*F*(2,105) = 4.27, *p* < 0.05), with no interaction between these factors detected (*F*(4,105) = 0.54, *p* = 0.71). Planned comparisons showed that both male and diestrus female rats displayed significantly higher levels of freezing compared to proestrus female rats across all three trials (*p* < 0.001; Fig. 3H). For flight scores, both group (*F*(2,105) = 110.11, *p* < 0.001) and trial (*F*(2,105) = 4.09, *p* < 0.05) effects were significant, without a detected interaction (*F*(4,105) = 0.55, *p* = 0.70). Subsequent planned comparisons revealed that proestrus female rats displayed significantly higher flight responses than both male and diestrus female rats on every trial (*p* < 0.001; Fig. 3I). We did not quantify fleeing and jumping behavior separately as jumping response was absent during the retention test. Taken together, our study, set in a larger arena with ecologically relevant stimuli, reveals distinct sex differences, particularly active defensive behaviors in proestrus females, underscoring the crucial role of hormones in shaping behavioral responses.

## Discussion

The present study found the critical role of the naturalistic predatory threats in mediating different types of defensive response, especially in female rats during specific stages of the estrous cycle. We examined two contrasting defensive behaviors, freezing and flight, to probe sex-dependent expressions of conditioned fear responses (CR). Consistent with a large number of previous studies, the conventional fear conditioning protocol with FUS elicited predominantly freezing CR^28–30^, with little observable difference between the sexes. Conversely, the employment of PUS enabled the observation of flight responses across all three groups. However, these responses were predominantly maintained in proestrus female rats. These findings suggest that the type of defensive response to a threat cue is modulated by sex and hormonal state, but these effects may only become apparent under naturalistic circumstances.

Our study demonstrated that a defensive flight response was reliably observed in proestrus female rats during both the acquisition and retrieval phases of fear conditioning with PUS. However, no such difference was found in conditioning with FUS. One obvious difference between the two experiments is the type of the US used, which might have played a critical role in differentiating the expression of the defensive response to the learned threat cue. According to the Predatory Imminence Continuum theory^19^, an influential theory of defensive behavior, states that the choice of defensive behavior is largely determined by the perceived proximity of the threat which includes both spatial and temporal distance. Interestingly, there was a 6.5-s difference in the interstimulus interval between the two threat conditions: 9 s for FUS compared to 2.5 s for PUS. This discrepancy hints at the potential for altered threat perception and subsequent choice of defensive behavior due to differing levels of temporal imminence. While our study provides initial insights, the influence of this temporal variation on threat perception warrants further investigation.

Another key determinant might be the spatial configuration of the environment where the animal is exposed to the threat^31–33^. The conditioning box, where FUS was presented, was mainly a trap-like space surrounded by walls as opposed to the spacious arena where PUS was utilized. From the rat’s perspective, the spacious arena offers an escape route, thereby promoting the choice of running as a viable defensive option. Therefore, when a threat is uncertain or an escape route is unavailable in a confined space, it is highly probable for rats to lean towards a more passive defensive form of response^18^. This might have been the case for FUS-based fear conditioning where dermal pain is not readily recognized as a threat by the innate defensive system. In such a context, the animal’s choice of defensive response is primarily immobility. In contrast, PUS-based fear conditioning, where threat imminence is perceptible, flight responses are observed more frequently during the early trials in all animals. Yet, as the trials progressed, the flight responses of male and diestrus female rats significantly decreased, shifting to the more passive freezing behavior. However, proestrus female rats maintained a high level of flight responses throughout the conditioning sessions. This suggests that the proestrus rats inherently prefer active defensive behavior in threat situations, possibly influenced by cyclical hormonal changes, which subsequently impact their motivation-to-action mapping.

The sexually dimorphic responses found in our study could be attributable to the differently wired defensive circuits in males and females. However, given that diestrus female rats and male rats demonstrated comparable freezing responses, it suggests these sex differences in defensive behaviors are not determined by hardwired circuitry. Instead, these differences became more pronounced during certain phases of the estrous cycle, indicating that female defensive behavior is likely due to transient neuronal activity differences induced by sex hormones. The active behavior observed in ovulating females are beneficial as it enhances their chances of encountering suitable mates and maximizing their reproductive success^34^, even in environments with potential dangers. For example, previous study reports that proestrus female rats exhibit reduced freezing and engage in active risk assessment when exposed to cat odor, while diestrus female rats display more passive freezing behavior^35^. In line with this, we observed that proestrus female rats traveled significantly more distance than diestrus female rats in the open field test (Figs. 2B, 3B). Furthermore, two forms of defensive responses, active and passive fear responses, have been identified^36^, which might be dissociable within the amygdala. In addition, the neuronal activity fluctuates across the estrous cycle within different regions in the amygdala^37^ providing further evidence for the involvement of sex hormones in shaping defensive response.

The current study is one of the first attempts to directly compare different types of threats, presenting a challenge in equating the level of threats. While the level of threat is a subjective state and cannot be directly measured, behavioral indices such as freezing—a widely-used measure in rodent research—can serve as a viable reference across a varying degree of threat situations^38^. We chose three pairings of CS–US at 0.6 mA based on prior research findings^36,39^. PUS, however, is constrained by the maximum speed of robot due to mechanical limitations, which is also shared by the natural predators. Although it was fast enough to produce a robust fear response, we found increasing the number of PUS trials to five repetitions was necessary for comparable freezing levels in male and diestrus female rats to those from FUS during retention (Figs. 2G, 3H). Another notable adjustment was the US duration. The mechanical action of PUS requires at least 1.5 s to fully rotate the donut maze. For instances where animals are positioned at a distance from the robot, at least one and a half rotations are necessary to effectively induce chasing-associated threat. Thus, 2.5 s was a minimum duration to produce effective threat situations.

In summary, our study highlights sex differences and hormonal regulation in defensive behavior. These differences were particularly evident when using ecologically relevant fear conditioning paradigms, such as a predator-like robot and the spatial configuration such as the availability of escape routes. The significance of threat type and the context within which the defensive response is expressed has been recognized for many decades but has rarely been addressed in formal experiments. The present study emphasizes the need for an attention to a new venue for investigating the species-specific defensive behavior and sex difference in motivation-to-action mapping.

## Materials and methods

### Animals

Seventy adult (6–7 weeks old) male and female Sprague Dawley rats obtained from Orient Bio and Koatech, Korea were used in this study. The rats were acclimated to the laboratory for at least two weeks and underwent daily examination of vaginal lavage for at least 10 days prior to the behavioral tests. Rats were group housed (two/cage) in a climate (21 ± 2°) and humidity (50%) controlled vivarium, and they were maintained on a 12-h light–dark cycle with access to food and water ad libitum. All the behavioral experiments were conducted during the dark phase of the cycle. Due to the absence of any surgical process, no anesthesia was used. At the end of the experiment, the animals were euthanized with CO_2_. The study was approved by the Korea University Institutional Animal Care and Use Committee. All experiments were conducted in accordance with the US National Institutes of Health Guide for the Care and Use of Laboratory Animals and conformed to ARRIVE guideline.

### Estrous cycle monitoring

To ensure appropriate estrous cycles for behavioral tests, females underwent daily examination of vaginal lavage^40,41^ for 10 days prior to the behavioral tests. During behavioral tests, examinations of vaginal lavage were performed 1 h before each behavioral test. The identification of each phase was based on the proportion of nucleated epithelial cells and leukocytes observed in the vaginal smear. To quantify these types of cells, an image of the collected vaginal cells on a glass slide was captured using a light microscope (Nikon Eclipse Ci, Nikon Corporation, Tokyo, Japan). The captured image was then utilized for cell counting analysis, and female rats were categorized as either proestrus or diestrus based on an open field test and a fear conditioning day. One female rat was excluded due to an unstable estrous cycle. Male rats underwent similar procedure daily except pipette insertion to match the level of stress. To match the level of stress the females experienced, male rats underwent similar daily procedure as the vaginal smear, but without undergoing the pipette insertion.

### Open field test

Proestrus and diestrus female rats along with male rats were subjected to an open field test to assess their general activity levels. Rats were individually placed in an open arena (80 × 80 × 30 cm) and allowed to freely move for 10 min. After the free exploration, rats were returned to their home cages. The arena was cleaned with a 70% ethanol solution between animals. General activity levels including distance traveled and velocity were recorded and analyzed offline by a video tracking software Ethovision (XT 16, Noldus Information Technology)^42^.

### Fear conditioning with FUS

The fear conditioning paradigm employed in this study was adapted and modified from previous studies^36,39^. Animals underwent habituation, fear conditioning, and retention in two identical clear Plexiglas boxes (25 × 30 × 40 cm) enclosed within a sound-attenuating cubicle with background noise (45 dB SPL) provided by a ventilation fan. During habituation and retention test, the boxes were covered with bedding materials and lit with white light. For conditioning, each box was illuminated with green light and floored with 16 stainless steel rods (0.5 cm in diameter, 1.5 cm apart) connected to a scrambled shock generator (Precision Animal Shocker, Coulbourn Instruments, Whitehall, PA). A tone (2 kHz, 80 dB, 10 s) as a conditioned stimulus (CS) was presented from a small speaker on the side of each box connected to a tone generator (Programmable Tone/Noise Generator, Coulbourn Instruments, Whitehall, PA) through an amplifier (CPA-200, Vascom, Korea). For unconditioned stimulus (US), a footshock (0.6 mA, 1 s) was delivered through the grid.

On the first day of conditioning, we conducted habituation where animals were allowed to freely explore the chamber for 10 min. On the next day, following a 2-min acclimation period, rats received 3 CS trials which co-terminated with a 1-s footshock, with an average inter-trial interval (ITI) of 120 s ranging from 110 to 130 s. Then 24 h later, to test retention of fear memory, the rats received 3 CS presentations with a 20-s ITI in the same context as on the habituation. A video camera was mounted beside each box to monitor and analyze behaviors of rats, and the chamber was thoroughly cleaned with 70% ethanol at the end of each experiment.

### Fear conditioning with PUS

A predator-like robot, modeled after designs from an earlier study^25^, was used as a US (PUS) to simulate the experience of being chased by a predator. Briefly, the robot used in this experiment was a box-shaped vehicle (15 × 26 × 35 cm) with four high traction rubber tires. The speed (0.7 m/s) was controlled by a Bluetooth^®^-based microcontroller with a custom-written program in Arduino (Arduino, USA), and the CS (2 kHz, 80 dB, 5 s) was generated by two 8-Ω speakers mounted in the front and back of the robot. The direction of the robot’s movement (clockwise/counterclockwise) was randomly determined with equal probability. Fear conditioning with chasing robot was conducted in an apparatus consisted of an acrylic donut-shaped structure, comprising a clear acrylic cylinder (24 cm in diameter) nested within a larger black acrylic cylinder (60 cm in diameter). This configuration formed a circular track with a width of approximately 18 cm and a wall height of 45 cm (Fig. 3C). To monitor and record behaviors, a camera was positioned above the maze, while three additional cameras were placed inside the inner cylinder to capture any vertical movements such as rearing and jumping from all angles. White noise at a level of 60 dB was played to mask any ambient noise. All experiments were conducted in a large soundproof chamber (180 cm × 150 cm × 220 cm).

Rats were first habituated to the donut maze with a floor covered with bedding materials and illuminated by green light. After a 10-min exploration period, the rats were returned to their home cages. Then on conditioning day, rats underwent 5 pairings of the tone and 2.5-s of robot chasing separated by an average ITI of 40 s ranging from 30 to 50 s. The maze on the conditioning day had a flat floor and was illuminated by indirect halogen lightning. On retention day, CS memory was tested in the identical maze setting as habituation day by presenting 3 CSs. At the end of each experiment, the maze was thoroughly cleaned with 70% ethanol. One male rat was excluded from the analysis because the corresponding video file was missing.

### Defensive behavior analysis

Movement velocities were analyzed offline using Ethovision software (XT 16, Noldus Information Technology)^42^. Freezing was automatically scored when the velocities were less than 0.8 cm/s for at least 1 s during the presentations of CSs^42,43^. Based on criteria from a prior study^6^, a rapid fleeing movement exceeding a velocity of 26 cm/s with a minimum inter-peak interval of 0.7 s was scored as flight response. The threshold velocities for distinguishing between freezing and fleeing were determined by comparing automatic and manual scoring of these behaviors using a sample dataset. Vertical movements, such as jumping, were manually scored by an experimenter who was blind to the groups. The flight score was calculated by adding 1 point for each instance of fleeing behavior and an additional point for each jump escape observed during the presentation of CSs.

### Statistical analysis

Statistical analyses were performed using a statistics software package (SPSS version 27, IBM SPSS, Armonk, NY). Behavioral results were analyzed using two-way repeated measures analysis of variance (ANOVA) that contained within-subjects variables (trial) and between-subjects factors (group). Group comparisons were also conducted with one-way ANOVA or Student’s *t* tests. When significant interactions were detected, post-hoc pairwise comparisons were conducted using Bonferroni correction. To compare the cell types among female rats, Student’s *t*-tests were performed. Pearson’s correlation coefficient was employed to analyze the relationship between behavioral indices (freezing, flight responses, and collisions). Two-tailed *p*-values of < 0.05 were considered statistically significant. Data were expressed as mean ± SEM.

### Supplementary Information

Supplementary Information.

Supplementary Video 1.

Supplementary Video 2.

Supplementary Video 3.

## Data Availability

The data generated in this study are available upon direct request to the corresponding author.
