# Supplementary material for: Conditioned flight response in female rats to naturalistic threat is estrous-cycle dependent
Source: Sci Rep. 2023 Nov 28;13:20988. doi: 10.1038/s41598-023-47591-x (PMC10684534; doi:10.1038/s41598-023-47591-x)
Supplement: Supplementary file 1 — Supplementary Information. [file 41598_2023_47591_MOESM1_ESM.docx]

**Supplementary Information**

**Conditioned flight response in female rats to naturalistic threat is estrous-cycle dependent**

Gyeong Hee Pyeon, Jaeyong Lee, Yong Sang Jo, and June-Seek Choi^*^

School of Psychology, Korea University, Seoul, Republic of Korea

**Supplementary Figures**


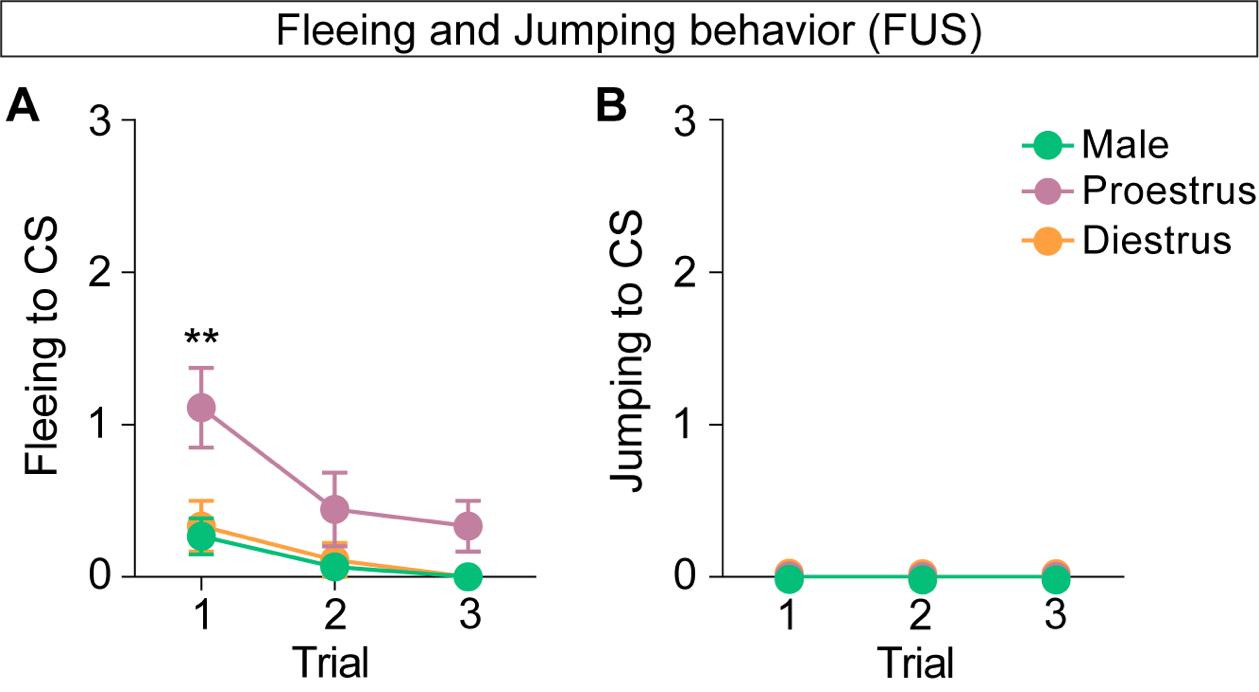


**Supplementary Fig. S1. Fleeing and jumping behavior to the CS during conditioning with FUS.** (A) Fleeing behavior to the CSs. There were both significant effects of group (*F*(2, 81) = 12.65, *p* < .001) and trial (*F*(2, 81) = 9.21, *p* < .001) in the number of fleeing, with no interaction observed (*F*(4, 81) = 1.25, *p* = .30). Planned comparisons revealed that proestrus female rats demonstrated significantly higher number of fleeing in response to the CS on trial 1 compared to both male and diestrus female rats (*p* < .01). (B) Jumping behavior to the CSs. None of the animals exhibited jumping behavior during conditioning with FUS.

**
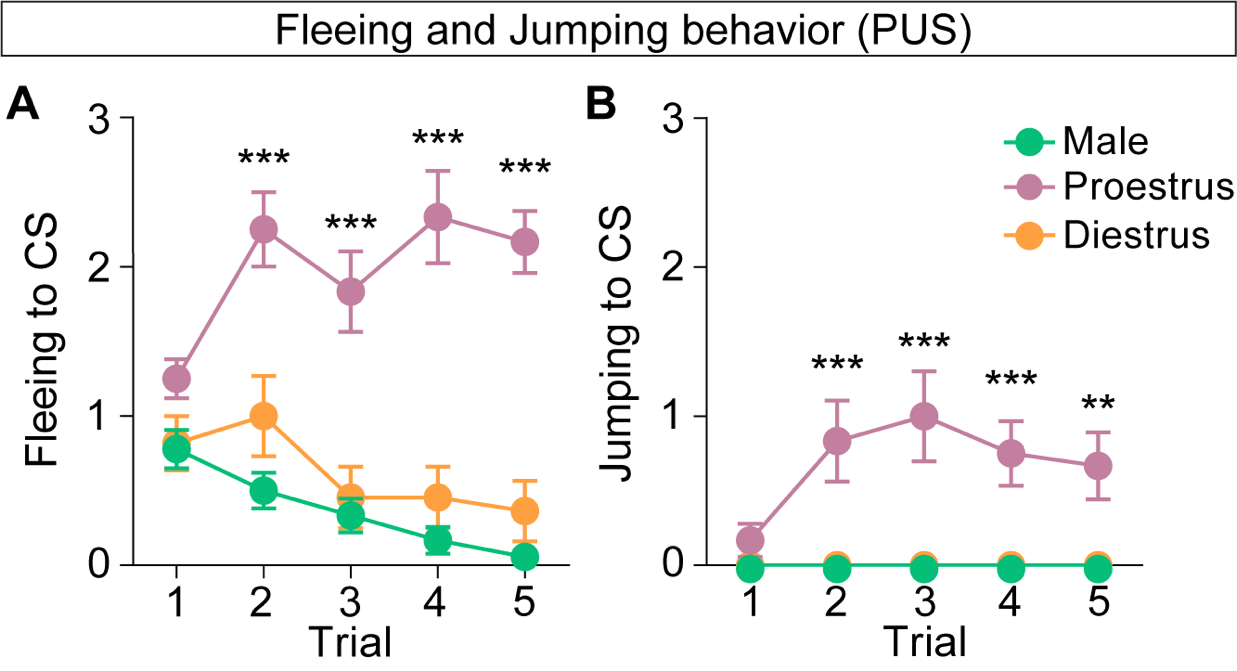
**

**Supplementary Fig. S2. Fleeing and jumping behavior to the CS during conditioning with PUS.**

(A) Fleeing behavior to the CSs. There was a significant group effect (*F*(2, 81) = 12.65, *p* < .001) and a marginal trial effect (*F*(2, 175) = 2.27, *p* = .063) on the number of fleeing. An interaction was also detected (*F*(8, 175) = 4.35, *p* < .001). Post hoc analyses revealed that, during trials 2 – 5, proestrus female rats demonstrated a significantly higher number of fleeing responses to the CS compared to both male and diestrus female rats (*p* < .001). (B) Jumping behavior to the CSs. Group differences were evident (*F*(2, 175) = 51.93, *p* < .001), alongside a marginal trial effect (*F*(2, 175) = 2.249, *p* = .065) and a significant interaction effect (*F*(8, 175) = 2.19, *p* < .05). Notably, while male and diestrus female rats did not display any jumping behavior, proestrus female rats exhibited a higher frequency of jumping behavior during trials 2 – 5 in response to the CSs (*p* < .01).

**Supplementary Videos**

**Supplementary Video S1.** Fear conditioning with PUS promotes conditioned freeing responses in male rats, Related to Figure 3.

**Supplementary Video S2.** Fear conditioning with PUS promotes conditioned flight responses in proestrus female rats.

**Supplementary Video S3.** Fear conditioning with PUS promotes conditioned freezing responses in diestrus female rats.
